# PTEN Expression as a Complementary Biomarker for Mismatch Repair Testing in Breast Cancer

**DOI:** 10.3390/ijms21041461

**Published:** 2020-02-21

**Authors:** Gianluca Lopez, Marianna Noale, Chiara Corti, Gabriella Gaudioso, Elham Sajjadi, Konstantinos Venetis, Donatella Gambini, Letterio Runza, Jole Costanza, Chiara Pesenti, Francesco Grossi, Stefania Maggi, Stefano Ferrero, Silvano Bosari, Nicola Fusco

**Affiliations:** 1 Division of Pathology, Fondazione IRCCS Ca’ Granda, Ospedale Maggiore Policlinico, 20122 Milan, Italy; gianluca.lopez@unimi.it (G.L.); gabriella.gaudioso@unimi.it (G.G.); elam.sajjadi@policlinico.mi.it (E.S.); letterio.runza@policlinico.mi.it (L.R.); stefano.ferrero@unimi.it (S.F.); 2 School of Pathology, University of Milan, 20122 Milan, Italy; 3 Neuroscience Institute Aging Branch, National Research Council (CNR), 35127 Padua, Italy; marianna.noale@in.cnr.it (M.N.); stefania.maggi@in.cnr.it (S.M.); 4 School of Medical Oncology, University of Milan, 20122 Milan, Italy; chiara.corti1@unimi.it; 5 Division of Pathology, IRCCS European Institute of Oncology (IEO), 20141 Milan, Italy; konstantinos.venetis@unimi.it; 6 Ph.D. School in Translational Medicine, University of Milan, 20122 Milan, Italy; 7 Division of Medical Oncology, Fondazione IRCCS Ca’ Granda, Ospedale Maggiore Policlinico, 20122 Milan, Italy; donatella.gambini@policlinico.mi.it (D.G.); francesco.grossi@policlinico.mi.it (F.G.); 8 Research Laboratories Coordination Unit, Fondazione IRCCS Ca’ Granda, Ospedale Maggiore Policlinico, 20122 Milan, Italy; jole.costanza@policlinico.mi.it; 9 Department of Oncology, IRCCS Mario Negri Institute for Pharmacological Research, 20156 Milan, Italy; chiara.pesenti@marionegri.it; 10 Department of Biomedical, Surgical and Dental Sciences, University of Milan, 20122 Milan, Italy

**Keywords:** breast cancer, mismatch repair, PTEN, immunohistochemistry, biomarkers, immunotherapy

## Abstract

Mismatch repair (MMR) analysis in breast cancer may help to inform immunotherapy decisions but it lacks breast-specific guidelines. Unlike in other neoplasms, MMR protein loss shows intra-tumor heterogeneity and it is not mirrored by microsatellite instability in the breast. Additional biomarkers can improve MMR clinical testing. Phosphatase and tensin homolog (PTEN) inactivation is an early oncogenic event that is associated with MMR deficiency (dMMR) in several tumors. Here, we sought to characterize the diagnostic utility of PTEN expression analysis for MMR status assessment in breast cancer. A total of 608 breast cancers were profiled for their MMR and PTEN status. Proteins expression and distribution were analyzed by immunohistochemistry (IHC) on tissue microarrays and confirmed on full sections; PTEN copy number alterations were detected using a real-time PCR assay. Overall, 78 (12.8%) cases were MMR-heterogeneous (hMMR), while all patterns of PTEN expression showed no intra-tumor heterogeneity. Wild-type PTEN expression was observed in 15 (18.5%) dMMR tumors (*p* < 0.0001). Survival analyses revealed significant correlations between MMR-proficient (pMMR), PTEN expression, and a better outcome. The positive predictive value of PTEN-retained status for pMMR ranged from 94.6% in estrogen receptor (ER)+/HER2- tumors to 100% in HER2-amplified and ER-/HER2- cases. We propose a novel diagnostic algorithm where PTEN expression analysis can be employed to identify pMMR breast cancers.

## 1. Introduction

The Food and Drug Administration (FDA) approval of the immune checkpoint inhibitor pembrolizumab in all refractory solid tumors with mismatch repair (MMR) deficiency has represented a paradigm shift in cancer therapy [1,2]. This indication is based on the concept that dysfunctions in the MMR system may result in genome instability [3,4]. The more mutations, the higher the chance that neo-antigens generated by the neoplastic cells will elicit the adaptative immune response, thus making the tumor likely sensitive to immunotherapy [5,6]. The two methods that are widely used to detect MMR deficiency in human cancers are immunohistochemistry (IHC) and sequencing of microsatellite loci [3]. The former is applied against four key MMR proteins (i.e., mutL homologue 1 (MLH1), mutS homologue 2 (MSH2), mutS homologue 6 (MSH6), and postmeiotic segregation increased 2 (PMS2)). Regrettably, no tumor-specific guidelines and/or companion diagnostic tests are available for MMR status assessment.

In breast cancers, alterations in the expression patterns of the MMR proteins are not exceptional, being observed at a frequency of 2–29%, and have both a prognostic and predictive value [7,8,9,10]. The pathological evaluation of MMR status, however, is controversial in these neoplasms [10,11]. Unlike in other types of tumors (e.g., those of the endometrium and colon-rectum), microsatellite instability (MSI) is restricted to a minority of breast cancers showing MMR protein loss [7,12]. Moreover, MMR IHC in breast cancer is troubled by a remarkable degree of intra-tumor heterogeneity in the expression of the MMR proteins [7]. Indeed, MMR-heterogeneous (hMMR) tumors may be mistakenly reported as MMR-deficient (dMMR) cases, if only the IHC-negative areas are sampled and/or analyzed. Given that hMMR and MMR-proficient (pMMR) breast cancers have an overlapping clinical course, a misdiagnosis might jeopardize the therapeutic approach. In this scenario, the identification of additional breast-specific biomarkers for MMR testing is required to improve the clinical management of these patients.

Phosphatase and tensin homolog (PTEN) is a key tumor suppressor that downregulates the phosphatidylinositol-3-kinase (PI3K)/protein kinase B (Akt) pathway and stimulates the expression of proapoptotic factors, preventing cell growth and survival [13]. Loss of PTEN activity has been identified in a wide spectrum of primary and metastatic neoplasms, including breast cancer [14]. This condition, which results in low or null expression of the protein, is an early oncogenic event [15,16]. In the context of the phase III Breast Cancer International Research Group (BCIRG) 006 trial investigating combination chemotherapy with or without trastuzumab, it has been observed that lack of PTEN expression by IHC is related to a poor prognosis in HER2-amplified tumors [17]. Recent data suggest that PTEN is also implicated in the MMR and overall DNA damage response in several types of tumors [18,19,20]. Regrettably, no specific information on the relationship between PTEN expression and MMR status in breast cancer have been published so far.

Our study aims to characterize the diagnostic potential of PTEN testing in breast cancer MMR status assessment. Here, we analyzed the MMR and PTEN status in a large series of breast cancers to define (i) the frequency, clinicopathologic features, and prognosis of PTEN-altered tumors; (ii) the impact of intra-tumor heterogeneity in PTEN testing; and (iii) the role of PTEN in MMR status prediction.

## 2. Results

### 2.1. Intra-Tumor Expression Patterns of PTEN and MMR Proteins

Among the 608 breast cancers analyzed, 328 (54%) cases showed retained PTEN expression (Table 1). The spatial distribution and IHC staining intensity of the protein were homogeneous across the different topographic areas of each tumor. Conversely, among the 159 (26%) neoplasms with loss of at least one of the MMR proteins, in approximately half of the cases (*n* = 78, 49%), IHC showed intra-tumor heterogeneity (Supplementary Table S2). In particular, heterogeneous loss of the MMR proteins was observed in 45 (7%), 52 (9%), 40 (7%), and 15 (2%) cases for MLH1, MSH2, MSH6, and PMS2, respectively (Supplementary Table S3). The highest prevalence of intra-tumor heterogeneity was observed for MSH2 in HER2-enriched breast cancers (*n* = 13/97, 13%). These observations suggest that the expression of PTEN, unlike that of the four MMR proteins, is homogeneous at a single-case level in breast cancer.

### 2.2. Clinicopathologic Features of PTEN-Low Breast Cancers

**Figure 1:**
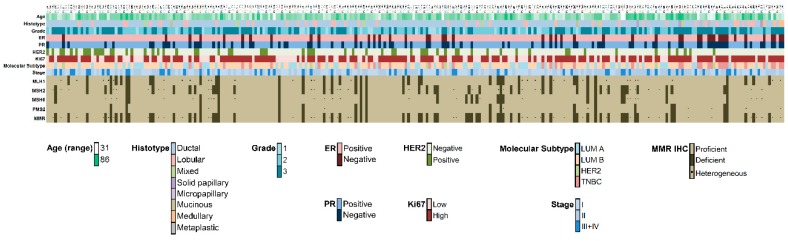
Overview of 280 PTEN-low breast carcinomas. Heatmap illustrating the clinical, histologic, and biological information together with mismatch repair protein status of all PTEN low (i.e., scores 0 and 1) cases identified. Each column represents a case, each row a parameter, which is color-coded according to the legend below. ER, estrogen receptor; PR, progesterone receptor; MLH1, mutL homologue 1; MSH2, mutS homologue 2; MSH6, mutS homologue 6; PMS2, postmeiotic segregation increased 2 (PMS2); MMR, mismatch repair; LUM A, Luminal A; LUM B, Luminal B; TNBC, triple-negative breast cancer; IHC, immunohistochemistry.

The mean age at diagnosis of the 280 patients with PTEN-low breast cancer was 62 years (range, 31–86 years), similar to that of PTEN-retained tumors (Table 1). The majority of PTEN-low tumors (*n* = 224, 80%) were invasive carcinomas of no special type (i.e., ductal) and encompassed 116 (41%) cases with lymph node metastasis (Figure 1 and Table 1). A higher proportion of HER2-enriched (*n* = 63, 23%) and estrogen receptor (ER)-/HER2- (*n* = 41, 15%) cases was observed in these tumors (*p* < 0.0001) with respect to PTEN-retained tumors (*n* = 34, 10% and *n* = 18, 5%, respectively), as detailed in Figure 1, Table 1 and Supplementary Table S3. Of note, PTEN-low breast cancers showed reduced expression of the MMR proteins, being classified as hMMR and dMMR in 40 (14%) and 66 (24%) cases, respectively (*p* < 0.0001). Somatic copy number alteration (SCNA) analysis of *PTEN* revealed that the majority (*n* = 51/66, 77%) of PTEN-low dMMR tumors harbored a reduced number of copies of the *PTEN* gene (Supplementary Table S4). Taken together, a low or null level of PTEN expression was more common in HER2-enriched and ER-/HER2- breast cancers and it was significantly related to higher levels of MMR deficiency.

### 2.3. The Prognostic Role of PTEN and MMR Status in Breast Cancers

Follow-up data were available for 603 (99%) patients. Bivariate analysis revealed a significant association between PTEN-retained expression and a lower death prevalence (*p* = 0.0001). Similar outcomes were observed in pMMR and hMMR tumors (*p* = 0.016) (Table 2), where survival analysis confirmed a better overall survival (Supplementary Figure S2).

These significant relationships were maintained in Luminal breast cancers (Supplementary Table S5) but not in ER- neoplasms (data not shown). Compared to cases harboring loss of PTEN, the progression-free survival of PTEN-retained tumors was significantly longer in Luminal A cancers (*p* = 0.01), as shown in Table 3.

According to Fisher’s exact test, the association between PTEN loss and disease relapse hazard ratio (HR) was significant in Luminal B breast cancers (*p* = 0.007), as represented in Table 4.

Loss of each of the MMR proteins, except for PMS2, was significantly associated with the disease-specific death in ER+ breast cancers (*p* < 0.05) (Supplementary Table S5). Taken together, loss of MSH2 alone was the most recurrent pattern of protein loss, being observed in 20 (25%) dMMR breast cancers (Supplementary Figure S3) and was related to shorter survival times (*p* = 0.04) (Supplementary Figure S4). Interestingly, the complete absence of expression of this protein, alone or in combination with the loss of MLH1, MSH6, and PMS2, was significantly associated with patients’ death, irrespective of the clinicopathological features of the tumors (*p* = 0.01), as displayed in Table 2.

### 2.4. Retained PTEN Expression Preferentially Identifies MMR-Proficient Breast Cancers

**Figure 2:**
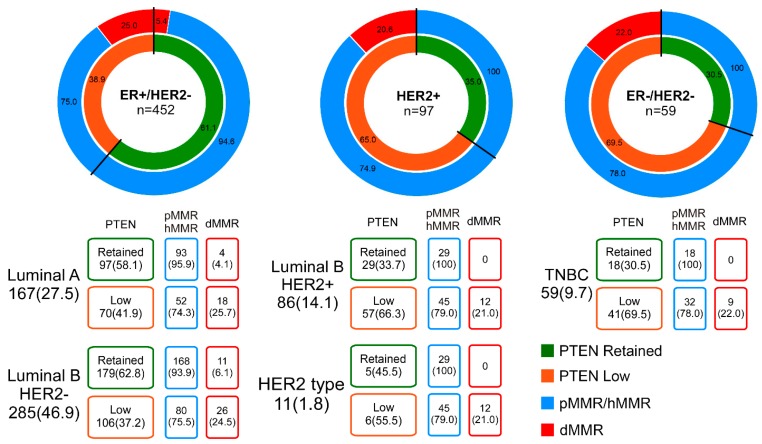
PTEN and mismatch repair protein expression across different biomarker-based subgroups of breast cancer patients. ER, estrogen receptor; TNBC, triple-negative breast cancer; pMMR, mismatch repair proficient; hMMR, mismatch repair heterogeneous; dMMR, mismatch repair deficient.

Analysis of PTEN according to the MMR status revealed that its retained expression is recurrent in breast cancers with an intact MMR system. Specifically, 95% (*n* = 313/328) PTEN-retained cases were found to be pMMR (*n* = 275) or hMMR (*n* = 38) (*p* < 0.0001), as represented in Table 1. Consistently, 82% (*n* = 66/81) of dMMR breast cancers showed low levels of PTEN expression and/or a decreased number of copies of the gene (Table 1, Supplementary Table S2). The association between PTEN and MMR status across the intrinsic molecular subtypes is portrayed in Figure 2.

In the ER+/HER2- cluster (*n* = 452), which encompasses both Luminal A (*n* = 167) and Luminal B HER2- (*n* = 285) breast cancers, a significant association of the MMR status according to PTEN IHC was observed (*p* < 0.0001, Fisher’s exact test) (Table 1 and Table 5).

In these patients, a PTEN-retained status by IHC showed a positive predictive value (PPV) for MMR proficiency of 94.6%. Remarkably, in HER2-enriched tumors (*n* = 97), including Luminal B HER2+ (*n* = 86) and HER2-type (*n* = 11) intrinsic molecular subtypes, and ER-/HER2− (*n* = 59) breast cancers, a highly significant association was observed. In both groups of patients, 100% of tumors with an intact MMR system showed a wild-type pattern of PTEN expression (*p* < 0.0001, Fisher’s exact test). These associations were significantly retained in a multinomial logistic model, as shown in Table 5. Taken together, the PPV for MMR proficiency of PTEN-retained IHC ranged from 95% to 100%, suggesting that PTEN analysis can help in MMR status assessment, overcoming the bias represented by MMR intra-tumor heterogeneity.

## 3. Discussion

The histology-agnostic approval of pembrolizumab represented a remarkable step forward in breast cancer treatment [21]. This unprecedented decision was based on 149 patients with MSI-high or dMMR cancers enrolled in five single-group clinical trials [2]. Among them, only 2/30 (6.7%) breast cancer patients from the phase Ib KEYNOTE-012 study completed the 2-year treatment and were of triple-negative (i.e., ER-negative, progesterone receptor (PR)-negative, and HER2-negative) phenotype [22]. In these studies, the trialists used locally developed tests (LDT) for patients’ selection. Therefore, no companion diagnostic test came with FDA approval. Whether the predictive diagnostic algorithms should be histology-agnostic remains a matter of controversy [23]. In this respect, the loss of the MMR proteins is not necessarily linked to an underlying MMR deficiency in breast neoplasms [7,24]. This specific biological trait has not been observed (or observed at lower frequencies) in tumors affecting other anatomical sites, such as lung cancer, melanoma, endometrial cancer, and colorectal cancer. Furthermore, MMR IHC and MSI analysis are not interchangeable tests in breast cancers, at least using the most commonly adopted diagnostic tools and criteria [7,12]. It remains to be determined the objective response rate of microsatellite-stable breast cancer patients showing loss of the MMR proteins when treated with an immune checkpoint blocker. Given that PTEN integrity is protective against MMR deficiency in several types of tumor [18,19,20], and that IHC is an excellent technique for identifying tumors with functional inactivation of this tumor suppressor [16,25,26], we have hypothesized that PTEN IHC can be of clinical value in MMR status assessment in breast cancers. Here, we performed a comprehensive analysis of a large series of non-familial breast cancers with long-term follow-up and found that, unlike for the MMR proteins, the expression of PTEN is homogeneous across the neoplasm. Furthermore, we confirmed that alterations in this tumor suppressor are more frequent in HER2-amplified and triple-negative breast cancers (TNBCs), being related to a worse prognosis. Finally, we demonstrated that a retained PTEN expression is strongly predictive for MMR proficiency.

To our knowledge, this is the first study aiming to define the role of PTEN as a complementary biomarker for MMR status assessment in breast cancer. The phenomenon of intra-tumor heterogeneity is a well-known major problem in breast cancer, particularly for biomarker-based treatment decision-making [27,28]. We confirm previous observation that the MMR proteins are heterogeneously expressed in an important proportion of breast cancers, with no preferential distribution inside the tumor area. Furthermore, hMMR breast cancers behave similarly to those that are pMMR, questioning the reliability of the MMR IHC analysis alone in identifying true dMMR breast cancers. The intrinsically low sensitivity of this testing method is obvious in small bioptic samples. Consistent with the crucial role of PTEN pathogenic alterations as founder genetic events in breast cancers, we provide previously unavailable evidence that the expression of PTEN is homogeneous across the tumor.

Loss of PTEN expression and MMR-related genomic instability are two of the most common molecular alterations in endometrial carcinoma [29] and they show a significant tendency toward co-occurrence [30]. However, it is still controversial as to whether there is a mechanistic relationship between these different molecular mechanisms [31,32,33,34]. It has been hypothesized that the polyadenosine tracts in *PTEN* might be a stochastic target for mutations in dMMR endometrial tumors [19,31,35,36]. Similar results were observed in colorectal cancer. The present analysis reveals that alterations in PTEN expression and SCNAs are more common in HER2-enriched and TNBCs compared to ER+/HER2- cases, being significantly related to MMR deficiency. In this regard, several studies have provided evidence to suggest that dMMR tumors exhibit a hypermutator phenotype, including *PTEN* somatic mutations [19,35,37].

**Figure 3:**
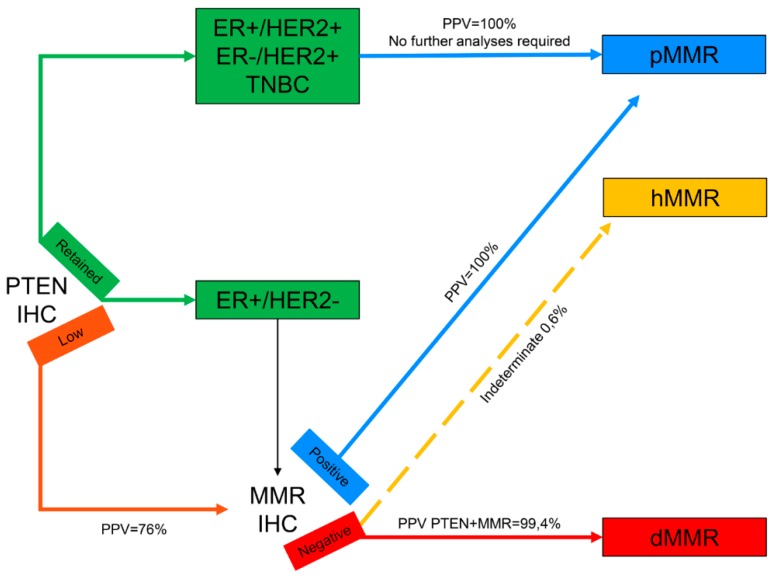
Revised diagnostic algorithm for the evaluation of mismatch repair status in breast cancer. Connectors drawn with continuous lines represent links between different steps of the diagnostic workflow; connectors drawn with discontinuous lines represent indeterminate biological characteristics. All connectors are color-coded based on the different biological features represented in the squared boxes. IHC, immunohistochemistry; ER, estrogen receptor; TNBC, triple-negative breast cancer; PPV, positive predictive value; MMR, mismatch repair; pMMR, mismatch repair proficient; hMMR, mismatch repair heterogeneous; dMMR, mismatch repair deficient.

In our analyses, retained PTEN expression by IHC emerged as a predictor of MMR proficiency, with PPVs ranging from 99.4% to 100% when performed upstream MMR IHC. Intriguingly, in ER+/HER2+ and ER-/HER2- breast cancers a retained IHC expression of PTEN allows for the identification of all pMMR tumors, making subsequent analyses not required. These observations allow for the delineation of a possible new diagnostic algorithm in breast cancer MMR status assessment (Figure 3). Thus, the implementation of PTEN IHC as a complementary diagnostic test in breast carcinomas is able to overcome MMR heterogeneity and its tremendous implications for treatment decision-making. It should be noted, however, that our data advocate that tumors with a PTEN-low phenotype and negative MMR IHC should not be classified as dMMR but as MMR-indeterminate, given that it is hardly ever possible to discern between hMMR and dMMR in this small subset of patients. For these cases, the identification of additional immune-related biomarkers would allow for more accurate patients’ stratification.

This proof-of-principle study has intrinsic limitations. First, we are aware that the frequency of dMMR breast cancers reported here is noticeably higher than that from massive parallel sequencing-based studies [38,39]. It should be noted, however, that the loss of only one protein was the most frequently observed pattern in our work (Supplementary Figure S3), involving 47% of dMMR neoplasms. We can posit that these unusual observations are not automatically linked to a hypermutator phenotype because the MMR complexes (i.e., MutL and MutS) are not entirely severed. On the other hand, these dMMR tumors have a significantly poorer prognosis. Further clinical studies coupled with centrally assessed companion diagnostic tests are warranted to investigate whether these patients would benefit from immune-checkpoint inhibition with pembrolizumab. Second, matched germlines were not examined to identify and subsequently exclude syndromic patients; however, the analysis of MLH1 promoter methylation, in addition to clinical and family information, is considered a rational Lynch syndrome testing surrogate [8,40,41]. Third, the use of tissue microarrays (TMAs) for the study of intra-tumor heterogeneity, albeit reliable, is not a gold standard; to reduce this possible drawback, the negative staining status was confirmed on full sections in all dMMR tumors. In addition, no data on the tumor-infiltrating lymphocytes, programmed death-ligand 1 (PD-L1) expression, and tumor mutational burden are provided here; then again, the approval of pembrolizumab in dMMR tumors does not take into account these features. Further translational studies encompassing extensive data on the tumor-intrinsic immunology are required to identify additional clinically relevant subclasses of breast cancer patients.

Despite these limitations, this study offers novel insights on PTEN IHC as a bona fide complementary diagnostic tool for immunotherapy, acting as a first-line screening test for the identification of pMMR breast cancers. The tumor-specific diagnostic algorithm proposed herein is a possible cost-effective tool for improving patients’ selection for immunotherapy. Our work lays the groundwork for the implementation of tailored MMR assays in the clinical workup of breast cancer patients.

## 4. Materials and Methods

This study is fully compliant with the local ethical guidelines and was approved by the Institutional Review Board (IRB) of Fondazione IRCCS Ca’ Granda – Ospedale Maggiore Policlinico under the protocol number #620_2018bis.

### 4.1. Patients and Tissue Specimens

A total of 608 patients with breast cancer (age, 26–92 years; mean ± standard deviation (SD), 61.0 ± 12.9 years) diagnosed and managed in the aforementioned Institution between 2004 and 2018 were included in this study (follow-up time, 1–172 months; mean ± SD, 57.8 ± 50.1 months). Their demographic and clinicopathologic characteristics are listed in Table 6. All cases are part of an institutional anonymized database encompassing detailed clinicopathologic data, including *MLH1* promoter methylation and MSI status, and regularly updated follow-up information [7]. Patients with a previous diagnosis and/or strong family history of breast, gynecological, and/or colorectal cancers, with pT1mi or pT1a breast tumors (i.e., <5 mm in greatest dimensions), likely syndromic according to the Revised Bethesda Guidelines for the identification of individuals at risk for Lynch syndrome (i.e., MLH1 IHC-negative MSI-high tumors showing no methylation of the *MLH1* promoter) [42] or who received neoadjuvant therapy were excluded. All cases were reviewed, re-classified, and re-graded according to the latest World Health Organization (WHO) recommendations [43] and the Nottingham histologic grading system [44], respectively. Pathologic re-staging was performed following the 8th edition of the American Joint Committee on Cancer (AJCC) Cancer Staging Manual [45]. The intrinsic molecular subtypes were assessed using the surrogate definitions proposed by the St. Gallen International Breast Cancer Conference panel [46].

### 4.2. Tissue Microarrays Construction

Representative formalin-fixed, paraffin-embedded (FFPE) tissue blocks of the cases included in the study were used to generate 17 TMAs containing 180 tumor cores each, with a total number of 3060 spots of tissue (mean of 5.8 tumor samples per patient; range, 2–7 samples). For each case, the sampling included both the core and periphery (i.e., invasive front) of the tumor, in situ (i.e., intraductal) component (if present), and matched normal epithelial breast tissue (i.e., glandular tissue with at least one non-neoplastic terminal ductal-lobular unit adjacent to the neoplasm). The TMA protocol was optimized for the IHC study of intra-tumor heterogeneity in FFPE archival tissue blocks of breast cancers [47].

### 4.3. Immunohistochemical Analysis

**Figure 4:**
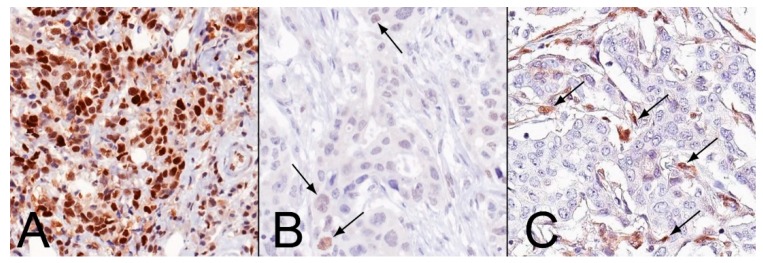
Patterns of MMR protein expression. (**A**). Proficient status in the presence of retained nuclear expression. (**B**). Heterogeneous expression where only a subset of neoplastic nuclei is positive (arrows). (**C**) Deficiency of the expression across the entire tumor in the presence of internal positive controls (arrows). Example of MLH1 immunohistochemistry; original magnification: 400×.

Four-μm-thick sections were cut from the TMA blocks and subjected to IHC using anti-human pre-diluted antibodies for ER, PR, Ki67, HER2, MLH1, MSH2, MSH6, PMS2, and PTEN on two automated staining systems (i.e., Dako Omnis, Agilent, Santa Clara, CA, USA; Ventana Benchmark Ultra, Roche, Switzerland) [7,48,49]. For each antibody, positive and negative controls were included in each slide run. The breast biomarkers (i.e., ER, PR, Ki-67, and HER2) were tested and reported according to the breast biomarker reporting guidelines v1.2.0.1 published by the College of American Pathologists (CAP) in August 2018 (available at https://www.cap.org/protocols-and-guidelines). The MMR status was analyzed separately in all distinct components, following previously reported criteria [50]. For each MMR protein, the loss of expression was defined by the complete absence of nuclear staining within all neoplastic cells [51]. Cancers showing retained expression of MLH1, MSH2, MSH6, and PMS2 were defined as pMMR, irrespective of the staining intensity. In the presence of internal positive control (i.e., tumor microenvironment cells and non-neoplastic epithelial cells from the terminal duct-lobular unit), the complete loss of at least one of these proteins across the entire tumor designated the dMMR status. When the protein was expressed only in a part of the tumor (i.e., <100% of tumor cells), the case was recorded as hMMR [7]. The three patterns of MMR protein expression are exemplified in Figure 4.

**Figure 5:**
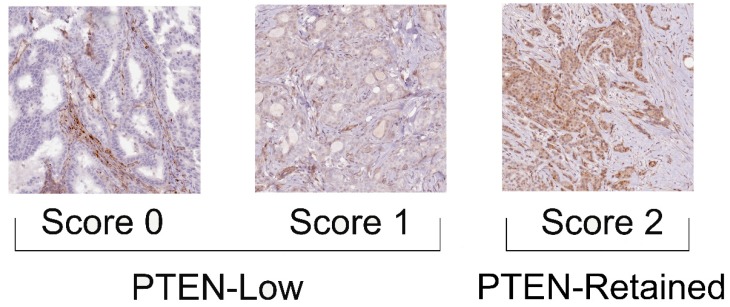
Patterns of PTEN expression and scoring system employed in the present study. Score 0 was assigned in the absence of immunohistochemical staining in the tumor cells; score 1 was assigned when the neoplastic cells showed a weaker staining than the normal counterpart; score 2 was assigned in cases with equal staining intensity between tumor and normal epithelial/stromal cells; Cases were clustered as PTEN-low (score 1 and score 0) and PTEN-retained (score 2). Original magnification: 100×.

Whole tissue tumor sections from all dMMR cases were analyzed to confirm that they were not hMMR. PTEN expression was scored using a three-tier system, where score 0 designated the absence of staining in tumor cells but not in the surrounding normal epithelial and stromal cells, score 1 was assigned for staining weaker than the surrounding normal epithelial and stromal cells, and score 2 in case of staining equal to that of the surrounding normal epithelial and stromal cells [49]. Subsequently, PTEN status was assessed semiquantitatively as PTEN-low (score 0 and score 1) and PTEN-retained (score 2), as depicted in Figure 5. The methods and scoring systems employed are detailed in Supplementary Table S1.

### 4.4. Cancer Cell Enrichment, DNA Extraction, and PTEN Copy Number Analysis

To detect *PTEN* SCNAs associated with MMR deficiency, we subjected all dMMR cases showing low or null levels of PTEN expression (i.e., scores 0 and 1) to *PTEN* copy number analysis. Representative 7-μm thick section of these tumors and matched non-neoplastic glandular breast tissue were stained with methylthioninium chloride (i.e., methylene blue) and manually microdissected using a sterile needle under a stereomicroscope (Optika SZO, Italy) [52]. This task was performed by two authors (G.L. and C.C.) under the supervision of a breast pathologist (N.F.). Next, genomic DNA was extracted [53]. To evaluate the SCNAs of *PTEN* and the adjacent loci, a real-time PCR assay was employed (TaqMan, Thermo Fisher Scientific, Waltham, MA, USA), as described previously [54]. Three different regions within the *PTEN* genomic locus (Chr.10q23.31) were targeted using Hs05098450_cn (Chr10: 87,873,820 on GRCh38), Hs05153578_cn (Chr10: 87,949,592 on GRCh38), and Hs05182682_cn (Chr10: 88,024,586 on GRCh38) TaqMan assays, as detailed in Supplementary Figure S1. The human ribonuclease P RNA component H1 gene (14q11.2) served as a reference target. For each sample, the *PTEN* targets and reference assays were simultaneously subjected to a triplex quantitative PCR, according to the manufacturer’s protocol. All experiments were performed in triplicate. For SCNAs quantification, instrument raw data were analyzed using the CopyCaller Software v2.1 (Thermo Fisher Scientific, Waltham, MA, USA). The number of copies of the target sequences in each sample was determined by relative quantitation using the comparative C_T_ method. This method measures the C_T_ difference (ΔC_T_) between target and reference sequences. The number of *PTEN* copies was then inferred as two times the relative quantity of targets compared to the reference.

### 4.5. Statistical Analysis

All statistical analyses were performed using the SAS 9.4 software (SAS Institute, Cary, NC, USA). Categorical variables were represented as the number and corresponding percentages of patients; continuous variables were summarized through the mean and SD or median and quartiles (Q1, Q3). The Shapiro-Wilk test was used to analyze the normal distributions of continuous variables [55]. Relationships between MMR status, PTEN expression, and the characteristics of the patient population (i.e., demographic and clinical traits, data on treatment, pathological, and molecular features) were assessed using Fisher’s exact test or Chi-squared test (categorical variables), and Wilcoxon rank-sum test (continuous variables) [56]. To identify factors associated with MMR deficiency, multinomial logistic regression models were defined considering a stepwise selection procedure (*p*-value to entry 0.25; *p*-value to stay 0.20). Odds ratio (OR) and corresponding 95% confidence interval (CI) were calculated for each variable. Cox’s proportional hazard regression analysis was performed to identify clinicopathologic factors associated with tumor progression by applying a purposeful selection of covariates [57]. The proportional hazard assumption of the Cox model was verified by the Schoenfeld’s residuals test. Quantitative variables were evaluated using the analysis of quartiles by linearity assumption [58]. For each predictor, the HR and corresponding 95% CI were calculated. Survival curves were built according to the Kaplan-Meier method and compared using the log-rank test [59]. All statistical tests were two-tailed; *p*-values < 0.05 were considered statistically significant.

## 5. Patents

The diagnostic algorithm and testing method proposed herein are subject of a patent application (#IT102018000010730).

## Figures and Tables

**Table 1:** Correlation between phosphatase and tensin (PTEN) and mismatch repair status across selected clinicopathologic features. SD, standard deviation; ER, estrogen receptor; MMR, mismatch repair.

	PTEN Low	PTEN Retained	*p*-Value
All patients, n (%)	280 (46.1)	328 (53.9)	
Age at diagnosis, mean ± SD	61.5 ± 12.0	60.6 ± 13.6	0.3425
Histological subtype, n (%)			0.6243
Ductal	224 (80.0)	267 (81.4)	
Lobular	40 (14.3)	39 (11.9)	
Other	16 (5.7)	22 (6.7)	
Clinical cluster, n (%)			<0.0001
ER+, HER2-	176 (62.9)	276 (84.2)	
HER2+	63 (22.5)	34 (10.4)	
ER-, HER2-	41 (14.6)	18 (5.5)	
Grade, n (%)			0.6428
1	34 (12.1)	39 (11.9)	
2	116 (41.4)	148 (45.1)	
3	130 (46.4)	141 (43.0)	
T, n (%)			0.7479
1	180 (64.3)	198 (60.4)	
2	83 (29.6)	105 (32.0)	
3	6 (2.1)	8 (2.4)	
4	11 (3.9)	17 (5.2)	
N, n (%)			0.2528
-	164 (58.6)	207 (63.1)	
+	116 (41.4)	121 (36.9)	
Stage, n (%)			0.4682
0, 1	118 (42.1)	154 (47.0)	
2	107 (38.2)	112 (34.2)	
3, 4	55 (19.6)	62 (18.9)	
MMR, n (%)			<0.0001
Proficient	174 (62.1)	275 (83.8)	
Deficient	66 (23.6)	15 (4.6)	
Heterogeneous	40 (14.3)	38 (11.6)	

**Table 2:** Bivariate analysis showing the association of selected clinicopathologic characteristics with patients’ death. ER, estrogen receptor; MMR, mismatch repair; MLH1, mutL homologue 1; MSH2, mutS homologue 2; MSH6, mutS homologue 6; PMS2, postmeiotic segregation increased 2 (PMS2).

	Death		*p*-Value
	Yes	No	
	(*n* = 34)	(*n* = 569)	
ER+/HER2, n (%)			0.004
ER+, HER2-	17 (4.0)	430 (96.0)	
HER2+	11 (11.3)	86 (88.7)	
ER-, HER2-	6 (10.2)	53 (89.8)	
Stage, n (%)			0.0006
0, 1	8 (3.0)	263 (97.0)	
2	12 (5.6)	204 (94.4)	
3, 4	15 (12.9)	101 (87.1)	
PTEN retained, *n* (%)	8 (2.5)	317 (97.5)	0.0001
MMR, *n* (%)			0.0161
Proficient or heterogeneous	25 (4.8)	499 (95.2)	
Deficient	10 (12.7)	69 (87.3)	
MLH1, *n* (%)			0.1712
Proficient or heterogeneous	30 (5.4)	528 (94.6)	
Deficient	5 (11.2)	40 (88.8)	
MSH2, *n* (%)			0.0107
Proficient or heterogeneous	27 (4.9)	520 (95.1)	
Deficient	8 (14.3)	48 (85.7)	
MSH6, *n* (%)			0.0641
Proficient or heterogeneous	31 (8.2)	545 (91.8)	
Deficient	4 (14.8)	23 (85.2)	
PMS2, *n* (%)			1
Proficient or heterogeneous	34 (5.8)	549 (94.2)	
Deficient	1 (5.0)	19 (95.0)	

**Table 3:** Bivariate analysis showing the association of selected clinicopathologic characteristics with tumor progression in Luminal A breast cancers. MLH1, mutL homologue 1; MSH2, mutS homologue 2; MSH6, mutS homologue 6; PMS2, postmeiotic segregation increased 2 (PMS2); MMR, mismatch repair.

	Progression		*p*-Value
	Yes	No	
	(*n* = 8)	(*n* = 150)	
Stage, *n* (%)			0.0346
0, 1	1 (1.3)	79 (98.7)	
2	5 (7.6)	61 (92.4)	
3, 4	2 (16.7)	10 (83.3)	
PTEN retained, n (%)	1 (1.1)	90 (98.9)	0.0106
MMR, n (%)			0.3084
Proficient or heterogeneous	6 (4.4)	130 (95.6)	
Deficient	2 (9.1)	20 (89.9)	
MLH1, n (%)			0.4462
Proficient or heterogeneous	7 (4.8)	140 (95.2)	
Deficient	1 (9.1)	10 (90.9)	
MSH2, n (%)			0.1687
Proficient or heterogeneous	6 (4.2)	137 (95.8)	
Deficient	2 (13.3)	13 (86.7)	
MSH6, n (%)			0.0416
Proficient or heterogeneous	6 (4.0)	145 (96.0)	
Deficient	2 (25.0)	6 (75.0)	
PMS2, n (%)			1
Proficient or heterogeneous	8 (5.2)	147 (94.8)	
Deficient	0	3 (100)	

**Table 4:** Clinicopathologic factors associated with tumor progression in Luminal B breast cancers. Model defined using a stepwise selection of the predictors; significance levels for entry (SLE = 0.25) and for stay (SLS = 0.20). NST, invasive carcinoma of no special type (ductal); HR, hazard ratio; CI, confidence interval.

	HR	95% CI	*p*-Value
Histological subtype, NST vs. other	0.39	0.14-1.06	0.0653
Systemic metastases	11.9	2.01–71.1	0.0064
Stage			
2 vs. (0, 1)	1.94	0.69-5.47	0.2377
(3, 4) vs. (0, 1)	3.56	1.29-9.81	0.0055
PTEN-low	3.24	1.37-7.65	0.0073
Lymphovascular invasion	2.31	0.85-6.29	0.1002

**Table 5:** Multivariable analysis showing the association of selected clinicopathologic characteristics and PTEN expression with MMR status. NST, invasive carcinoma of no special type (ductal); ILC, invasive lobular carcinoma; TNBC, triple-negative breast cancer; OR, odds ratio; CI, confidence interval; dMMR, mismatch repair-deficient; pMMR, mismatch repair proficient; hMMR, mismatch repair heterogeneous.

	dMMR vs. pMMR			hMMR vs. pMMR		
	OR	95% CI	*p*-value	OR	95% CI	*p*-value
Histological subtype						
NST vs. other	0.82	0.28–2.44	0.7235	0.48	0.20–1.12	0.1088
ILC vs. other	0.46	0.13–1.68	0.238	0.25	0.07–0.85	0.0262
Intrinsic molecular subtype						
Luminal B HER2− vs. Luminal A	2.35	0.91–6.05	0.078	0.76	0.22–2.59	0.662
Luminal B HER2+ vs. Luminal A	1.37	0.49–3.85	0.5495	1.02	0.29–3.59	0.9733
HER2-type vs. Luminal A	0.87	0.09–8.79	0.9034	0.89	0.13–6.33	0.9079
TNBC vs. Luminal A	1.41	0.41–4.84	0.5843	0.63	0.15–2.69	0.5279
Ki-67 (high vs. low)	0.3	0.12–0.73	0.0078	0.89	0.28–2.81	0.837
Grade						
2 vs. 1	2.13	0.84–5.37	0.1106	1.85	0.71–4.80	0.2051
3 vs. 1	2.94	1.09–7.89	0.0326	3	1.09–8.24	0.0333
PTEN (low vs. retained)	0.13	0.07–0.24	<0.0001	0.59	0.36–0.99	0.0435

**Table 6:** Clinicopathologic features of the patients included in this study according to their biomarker status. All cases were re-classified, re-graded, and re-assessed for hormone receptor, Ki67, and HER2 status according to the latest guidelines. NST, no special type; TNBC, triple-negative breast cancer; *ER+/PR+/Ki67 low; ^#^ER+/Ki67 high or ER+/PR-; ^∞^ER-/PR-/HER2+; ^§^ER-/PR-/HER2-.

	ER+/HER2-	HER2+	ER-/HER2-	Total
All patients, *n* (%)	452 (74.3)	97 (16.0)	59 (9.7)	608 (100)
Age at diagnosis, *n* (%)				
≥55 years	322 (76.1)	72 (17.0)	29 (6.9)	423 (69.6)
<55 years	130 (70.3)	25 (13.5)	30 (16.2)	185 (30.4)
Menopause, *n* (%)				
Yes	354 (75.0)	82 (17.4)	36 (7.6)	472 (77.6)
No	98 (72.1)	15 (11.0)	23 (16.9)	136 (22.4)
Histological subtype, *n* (%)				
Invasive carcinoma, NST	354 (72.1)	90 (18.3)	47 (9.6)	491 (80.7)
Lobular	74 (93.7)	5 (6.3)	0	79 (13.0)
Other	24 (63.2)	2 (5.3)	12 (31.5)	38 (6.3)
Histological grade, *n* (%)				
1	65 (89.0)	6 (8.2)	2 (2.7)	73 (12.0)
2	233 (88.3)	27 (10.2)	4 (1.5)	264 (43.4)
3	154 (56.8)	64 (23.6)	53 (19.6)	271 (44.6)
ER status, *n* (%)				
Positive	452 (84.0)	86 (16.0)	0	538 (88.4)
Negative	0	11 (15.7)	59 (84.3)	70 (11.6)
PR status, *n* (%)				
Positive	398 (85.2)	67 (14.3)	2 (0.4)	467 (76.8)
Negative	54 (38.3)	30 (21.2)	57 (40.4)	141 (23.2)
HER2 status, *n* (%)				
Positive	0	97 (100)	0	97 (16.0)
Negative	452 (88.5)	0	59 (11.5)	511 (84.0)
Ki67 status, *n* (%)				
High	262 (65.2)	83 (20.6)	57 (14.2)	402 (66.1)
Low	190 (92.2)	14 (6.8)	2 (1.0)	206 (33.9)
Stage, *n* (%)				
I	212 (77.9)	40 (14.7)	20 (7.4)	272 (44.7)
II	164 (74.9)	28 (12.8)	27 (12.3)	219 (36.0)
III-IV	76 (65.0)	29 (24.7)	12 (10.3)	117 (19.2)
Intrinsic molecular subtypes, *n* (%)				
Luminal A*	167 (100)	0	0	167 (27.4)
Luminal B^#^	285 (76.8)	86 (23.2)	0	371 (61.0)
HER2-type^∞^	0	11 (100)	0	11 (1.8)
TNBC^§^	0	0	59 (100)	59 (9.7)

## Supplementary Materials

Supplementary Materials can be found at https://www.mdpi.com/1422-0067/21/4/1461/s1.

## Abbreviations

FDA	Food and Drug Administration
MMR	mismatch repair
IHC	immunohistochemistry
MLH1	mutL homologue 1
MSH2	mutS homologue 2
MSH6	mutS homologue 6
PMS2	postmeiotic segregation increased 2
MSI	Microsatellite instability
hMMR	MMR-heterogeneous
dMMR	MMR-deficient
pMMR	MMR-proficient
PTEN	Phosphatase and tensin homolog
PI3K	phosphatidylinositol-3-kinase
Akt	protein kinase B
BCIRG	Breast Cancer International Research Group
ER	estrogen receptor
SCNA	somatic copy number alteration
HR	hazard ratio
PPV	positive predictive value
PR	progesterone receptor
LDT	locally developed tests
TNBCs	triple-negative breast cancers
TMAs	tissue microarrays
PD-L1	programmed death-ligand 1
IRB	Institutional Review Board
SD	standard deviation
WHO	World Health Organization
AJCC	American Joint Committee on Cancer
FFPE	formalin-fixed, paraffin-embedded
CAP	College of American Pathologists
OR	odds ratio
CI	confidence interval
