# Supplementary material for: PTEN Expression as a Complementary Biomarker for Mismatch Repair Testing in Breast Cancer
Source: Int J Mol Sci. 2020 Feb 21;21(4):1461. doi: 10.3390/ijms21041461 (PMC7073136; doi:10.3390/ijms21041461)
Supplement: Supplementary file 1 [file ijms-21-01461-s001.zip › Lopez et al - Int J Mol Sci - Supplementary Tables.docx]

| **Marker** | **Clone** | **Dilution** | **Technology** | **Antigen retrieval** | **Scoring** |
| --- | --- | --- | --- | --- | --- |
| ER | EP1 | RTU | Dako Omnis | EnVision FLEX, High pH, 20' | ASCO/CAP and St Gallen guidelines; positive if ≥1% of tumor cell nuclei are immunoreactive, high if >20% of tumor cell nuclei are immunoreactive |
| PR | PgR 636 | 1:100 | Dako Omnis | EnVision FLEX, High pH, 30’ | ASCO/CAP and St Gallen guidelines; positive if ≥1% of tumor cell nuclei are immunoreactive, high if >20% of tumor cell nuclei are immunoreactive |
| Ki67 | MIB1 | RTU | Dako Omnis | EnVision FLEX, High pH, 30’ | ASCO/CAP and St Gallen guidelines; high if >20% of tumor cell nuclei are immunoreactive |
| HER2 | Polyclonal | 1:400 | Dako Omnis | EnVision FLEX, Low pH, 30' | ASCO/CAP guidelines; 3+ if uniform intense membrane staining circumferential membrane staining that is complete and intense, 2+ if circumferential membrane staining that is incomplete and/or weak/moderate and within >10% of the invasive tumor cells or complete and circumferential membrane staining that is intense and within ≤10% of the invasive tumor cells, negative for other staining patterns |
| MLH1 | ES05 | 1:50 | Ventana Benchmark Ultra | CC1, 68’ | Negative if complete loss of nuclear staining within all tumor cells |
| MSH2 | FE11 | 1:50 | Ventana Benchmark Ultra | CC1, 68’ | Negative if complete loss of nuclear staining within all tumor cells |
| MSH6 | EP49 | 1:100 | Ventana Benchmark Ultra | CC1, 68’ | Negative if complete loss of nuclear staining within all tumor cells |
| PMS2 | EP51 | 1:50 | Ventana Benchmark Ultra | CC1, 68’ | Negative if complete loss of nuclear staining within all tumor cells |
| PTEN | 6H2.1 | 1:100 | Dako Omnis | EnVision FLEX, High pH, 30' | Retained expression: score 2 (staining in tumor cells equal to normal ductal and stromal cells);  Low expression: score 1 (staining in tumor cells weaker than normal ductal and stromal cells) or score 0 (staining absent in tumor cells but present in normal ductal and stromal cells). |
| **Supplementary Table S1. List of antibodies, clones, dilutions, antigen retrieval methods, and scoring systems adopted for immunohistochemical analyses.** ER, estrogen receptor alpha; PR, progesterone receptor; MLH1, MutL homolog 1; MSH2, MutS homolog 2; MSH6, MutS homolog 6; PMS2, PMS1 Homolog 2; PTEN, Phosphatase and tensin homolog; RTU, ready to use. | | | | | |

|  | **Mismatch repair status** | | | |
| --- | --- | --- | --- | --- |
|  | **Deficient** | **Proficient** | **Heterogeneous** | **p-value** |
| All patients, n (%) | 81 (13.3) | 449 (73.9) | 78 (12.8) |  |
| Age at diagnosis, median years ±SD | 63.5±12.3 | 60.6±12.7 | 61.0±14.3 | 0.1586 |
| Histological subtype, n (%)  Invasive carcinoma, NST  Lobular  Others | 66 (81.5)  10 (12.4)  5 (6.2) | 361 (80.4)  63 (14.0)  25 (5.6) | 64 (82.1)  6 (7.7)  8 (10.3) | 0.3483 |
| ER+HER2, n (%)  ER+, HER2-  HER2+  ER-, HER2- | 59 (72.8)  13 (16.1)  9 (11.1) | 341 (76.0)  67 (14.9)  41 (9.1) | 52 (66.7)  17 (21.8)  9 (11.5) | 0.5005 |
| Grade, n (%)  1  2  3 | 7 (8.6)  36 (44.4)  38 (46.9) | 60 (13.4)  199 (44.3)  190 (42.3) | 6 (7.7)  29 (37.2)  43 (55.1) | 0.1972 |
| T, n (%)  1  2  3  4 | 42 (51.9)  31 (38.3)  2 (2.5)  6 (7.4) | 291 (64.8)  130 (29.0)  10 (2.2)  18 (4.0) | 45 (57.7)  27 (34.6)  2 (2.6)  4 (5.1) | 0.3923 |
| N, n (%)  -  + | 41 (50.6)  40 (49.4) | 283 (63.0)  166 (37.0) | 47 (60.3)  31 (39.7) | 0.1289 |
| Stage, n (%)  0, 1  2  3, 4 | 28 (34.6)  34 (42.0)  19 (23.5) | 211 (47.0)  159 (35.4)  79 (17.6) | 33 (42.3)  26 (33.3)  19 (24.4) | 0.2046 |
| PTEN, n (%)  Retained  Low | 15 (18.5)  66 (81.5) | 275 (61.3)  174 (38.7) | 38 (48.7)  40 (51.3) | <0.0001 |
| **Supplementary Table S2. Clinicopathologic features of the patients included in this study according to their mismatch repair status**. All cases were re-classified, re-graded, and re-assessed for hormone receptor, Ki67, and HER2 status according the latest guidelines. NST, no special type. | | | | |

|  | **Total (%)**  **N=608** | **ER+/HER2-**  **N=452** | | **HER2+**  **N=97** | | **ER-/HER2-**  **N=59** |
| --- | --- | --- | --- | --- | --- | --- |
| MLH1 |  |  |  | |  | |
| Proficient | 518 (86) | 394 (87) | 78 (80) | | 46 (78) | |
| Deficient | 45 (7) | 29 (6) | 9 (9) | | 7 (12) | |
| Heterogeneous | 45 (7) | 29 (6) | 10 (10) | | 6 (10) | |
| MSH2 |  |  |  | |  | |
| Proficient | 500 (8) | 376 (83) | 73 (75) | | 51 (86) | |
| Deficient | 56 (9) | 39 (9) | 11 (11) | | 6 (10) | |
| Heterogeneous | 52 (9) | 37 (8) | 13 (13) | | 2 (3) | |
| MSH6 |  |  |  | |  | |
| Proficient | 541 (89) | 399 (88) | 85 (88) | | 57 (97) | |
| Deficient | 27 (4) | 19 (4) | 6 (6) | | 2 (3) | |
| Heterogeneous | 40 (7) | 34 (8) | 6 (6) | | 0 | |
| PMS2 |  |  |  | |  | |
| Proficient | 571 (94) | 431 (95) | 90 (93) | | 50 (85) | |
| Deficient | 22 (4) | 11 (2) | 6 (6) | | 5 (8) | |
| Heterogeneous | 15 (2) | 10 (2) | 1 (1) | | 4 (7) | |
| PTEN |  |  |  | |  | |
| Retained | 328 (54) | 276 (61) | 34 (35) | | 18 (31) | |
| Low | 280 (46) | 176 (39) | 63 (65) | | 41 (69) | |
| **Supplementary Table S3. Mismatch repair and PTEN protein status assessed by immunohistochemistry according to the three clinical clusters of breast cancer**. | | | | | | |

| **ID** | **cCN1** | **cCN2** | **cCN3** | ***PTEN* CN** |
| --- | --- | --- | --- | --- |
| BR007 | 2.50 | 2.28 | 2.05 | 2 |
| BR016 | 2.07 | 1.87 | 1.74 | 2 |
| BR026 | 0.80 | 1.06 | 1.13 | 1 |
| BR038 | 2.62 | 2.84 | 2.79 | 2 |
| BR040 | 0.91 | 1.36 | 1.33 | 1 |
| BR043 | 1.81 | 1.92 | 1.29 | 1 |
| BR045 | 1.16 | 1.54 | 1.48 | 1 |
| BR048 | 0.98 | 1.15 | 1.11 | 1 |
| BR049 | 1.70 | 1.60 | 1.81 | 1 |
| BR067 | 2.60 | 2.87 | 3.18 | 2 |
| BR069 | 1.80 | 1.84 | 1.80 | 1 |
| BR082 | 0.09 | 0.43 | 0.32 | 0 |
| BR093 | 2.21 | 2.83 | 2.96 | 2 |
| BR095 | 0.97 | 1.03 | 0.94 | 1 |
| BR098 | 0.98 | 1.40 | 1.10 | 1 |
| BR104 | 0.34 | 1.00 | 0.87 | 1 |
| BR105 | 0.13 | 0.60 | 0.50 | 0 |
| BR107 | 0.76 | 1.22 | 1.00 | 1 |
| BR113 | 0.23 | 0.82 | 0.78 | 0 |
| BR149 | 1.81 | 2.00 | 1.40 | 1 |
| BR161 | 0.39 | 1.18 | 1.00 | 1 |
| BR174 | 0.29 | 1.03 | 0.96 | 1 |
| BR177 | 0.29 | 0.90 | 0.74 | 1 |
| BR180 | 0.57 | 0.71 | 0.75 | 1 |
| BR186 | 1.30 | 1.43 | 1.00 | 1 |
| BR188 | 1.66 | 1.40 | 1.77 | 1 |
| BR192 | 2.15 | 1.80 | 2.00 | 2 |
| BR208 | 1.23 | 1.43 | 1.08 | 1 |
| BR210 | 0.15 | 0.61 | 0.48 | 0 |
| BR214 | 0.66 | 1.30 | 0.96 | 1 |
| BR215 | 0.69 | 0.82 | 0.80 | 1 |
| BR218 | 1.39 | 1.85 | 1.84 | 2 |
| BR222 | 1.41 | 1.56 | 1.78 | 2 |
| BR223 | 0.73 | 0.75 | 0.77 | 1 |
| BR239 | 0.70 | 1.20 | 1.09 | 1 |
| BR240 | 0.54 | 0.74 | 0.83 | 1 |
| BR241 | 0.97 | 1.13 | 1.20 | 1 |
| BR243 | 1.25 | 1.36 | 1.43 | 1 |
| BR244 | 0.28 | 0.73 | 0.55 | 1 |
| BR248 | 1.88 | 1.69 | 1.70 | 2 |
| BR251 | 0.75 | 0.85 | 0.80 | 1 |
| BR252 | null | 0.34 | 0.47 | 0 |
| BR253 | 0.47 | 0.76 | 0.93 | 1 |
| BR259 | 0.92 | 1.11 | 1.23 | 1 |
| BR265 | null | 0.58 | 0.56 | 1 |
| BR281 | 0.62 | 1.19 | 1.09 | 1 |
| BR285 | 0.11 | 0.45 | 0.37 | 0 |
| BR297 | 1.44 | 1.63 | 1.52 | 2 |
| BR298 | 1.24 | 1.39 | 1.38 | 1 |
| BR302 | 0.89 | 1.40 | 1.40 | 1 |
| BR306 | 0.99 | 1.05 | 1.38 | 1 |
| BR311 | 3.95 | 3.08 | 3.63 | 3 |
| BR316 | 2.05 | 2.17 | 1.80 | 2 |
| BR325 | 1.05 | 1.32 | 1.27 | 1 |
| BR326 | 1.93 | 2.10 | 1.87 | 2 |
| BR328 | 1.91 | 1.96 | 1.86 | 2 |
| BR334 | 2.53 | 2.34 | 2.27 | 2 |
| BR337 | 1.00 | 1.20 | 1.40 | 1 |
| BR356 | 1.70 | 1.80 | 1.79 | 1 |
| BR358 | 0.73 | 0.75 | 0.77 | 1 |
| BR363 | 1.60 | 1.10 | 1.09 | 1 |
| BR411 | 0.60 | 1.30 | 1.10 | 1 |
| BR417 | 1.10 | 1.20 | 1.10 | 1 |
| BR474 | 1.25 | 1.40 | 1.10 | 1 |
| BR490 | 1.30 | 0.20 | 0.55 | 1 |
| BR532 | 0.28 | 0.90 | 1.00 | 1 |
| **Supplementary Table S4. PTEN copy number analysis in 66 dMMR breast cancers showing low or null levels of PTEN protein expression by immunohistochemistry.** Three genomic regions were investigated, namely cCN1-Hs05098450_cn (Chr10: 87873820), cCN2-Hs05153578_cn (Chr10: 87949592), and cCN3-Hs05182682_cn (Chr10: 88024586). CN, copy numbers. | | | | |

|  | **Death** | | **p-value** |
| --- | --- | --- | --- |
|  | **Yes (n=27)** | **No (n=506)** |  |
| Molecular subtype, n (%)  Luminal A  Luminal B HER2-  Luminal B HER2+ | 4 (2.5)  14 (5.3)  9 (11.7) | 163 (97.5)  266 (94.7)  77 (88.3) | 0.0214 |
| Stage, n (%)  0, 1  2  3, 4 | 6 (2.5)  10 (5.6)  11 (12.4) | 241 (97.5)  176 (94.4)  89 (87.6) | 0.0042 |
| PTEN retained, n (%) | 7 (2.4) | 295 (97.6) | 0.0001 |
| MMR, n (%)  Proficient or heterogeneous  Deficient | 17 (3.8)  10 (17.0) | 447 (96.2)  59 (83.0) | 0.0010 |
| MLH1, n (%)  Proficient or heterogeneous  Deficient | 22 (4.7)  5 (15.2) | 473 (95.3)  33 (84.8) | 0.0356 |
| MSH2, n (%)  Proficient or heterogeneous  Deficient | 19 (4.1)  8 (19.5) | 465 (95.9)  41 (80.5) | 0.0017 |
| MSH6, n (%)  Proficient or heterogeneous  Deficient | 23 (4.7)  4 (19.1) | 485 (95.3)  21 (80.9) | 0.0318 |
| PMS2, n (%)  Proficient or heterogeneous  Deficient | 26 (5.3)  1 (7.1) | 492 (94.7)  14 (92.9) | 0.5464 |
| **Supplementary Table S5. Bivariate analysis showing the association of selected clinicopathologic characteristics with patients’ death in Luminal breast cancers with available follow-up data (n=533).** MMR, mismatch repair. | | | |
