# Supplementary figures and images for: PTEN Expression as a Complementary Biomarker for Mismatch Repair Testing in Breast Cancer
Source: Int J Mol Sci. 2020 Feb 21;21(4):1461. doi: 10.3390/ijms21041461 (PMC7073136; doi:10.3390/ijms21041461)

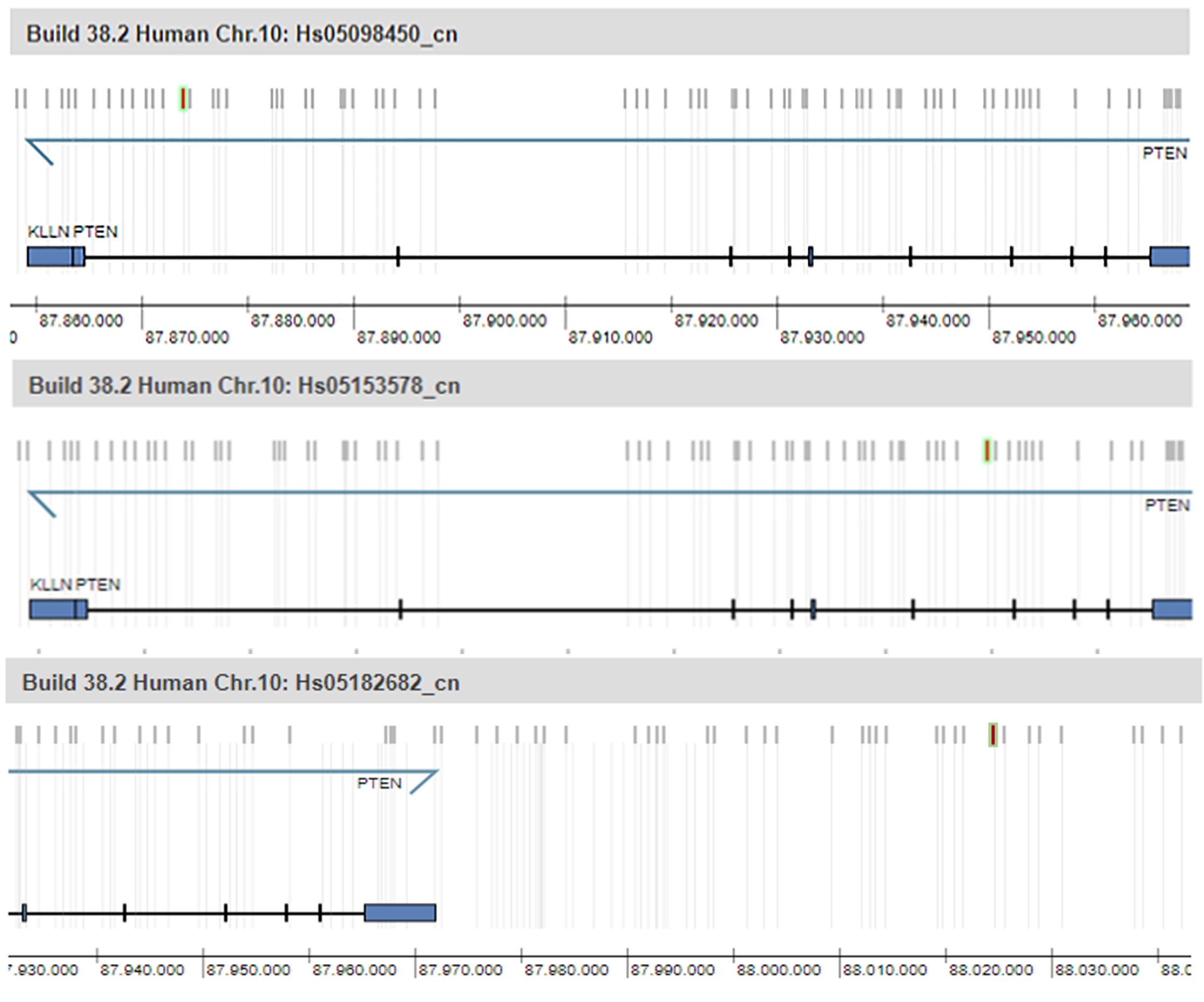

Supplement: Supplementary file 1 [file ijms-21-01461-s001.zip › Lopez et al - Int J Mol Sci - Supplementary Figure S1.tif]

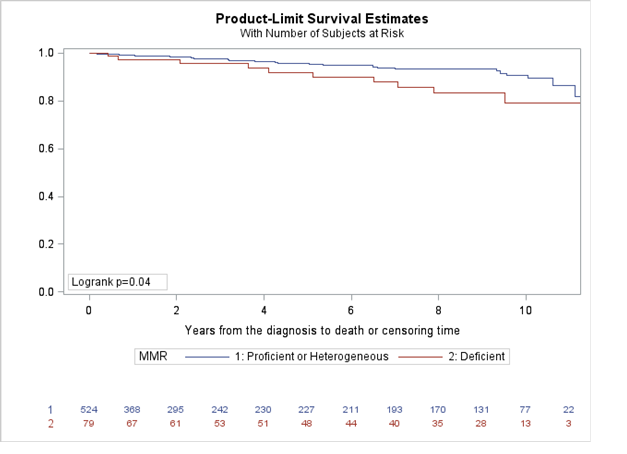

Supplement: Supplementary file 1 [file ijms-21-01461-s001.zip › Lopez et al - Int J Mol Sci - Supplementary Figure S2.tif]

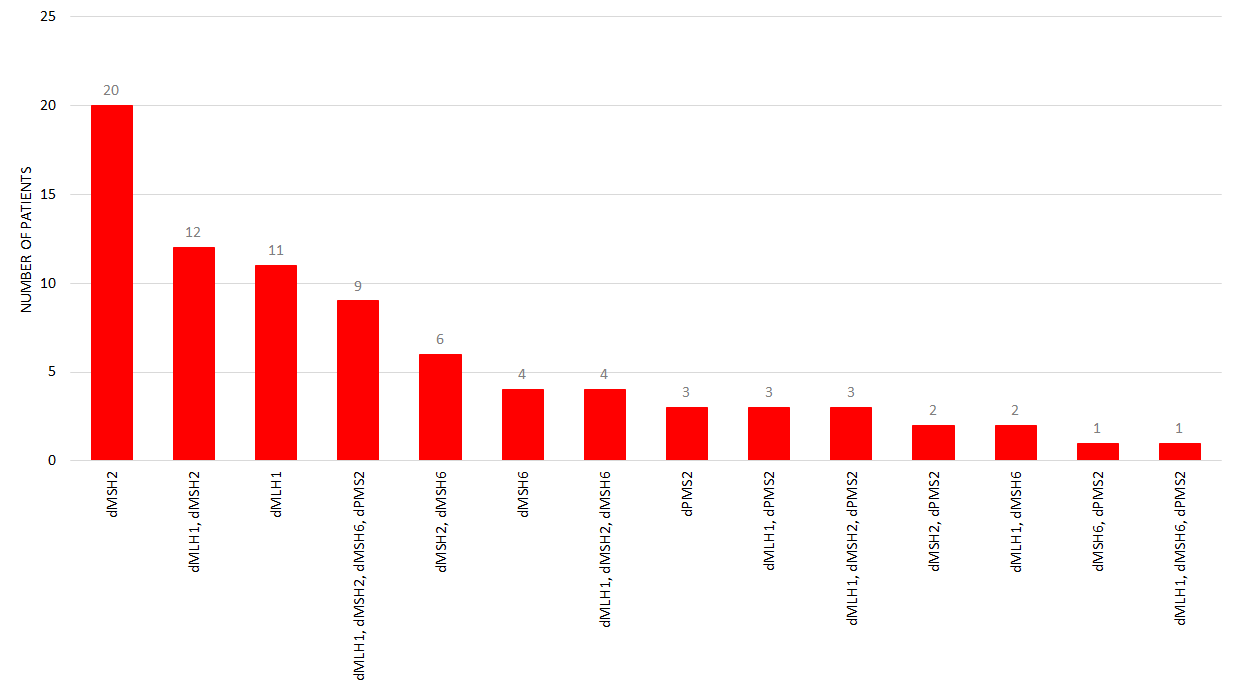

Supplement: Supplementary file 1 [file ijms-21-01461-s001.zip › Lopez et al - Int J Mol Sci - Supplementary Figure S3.tif]

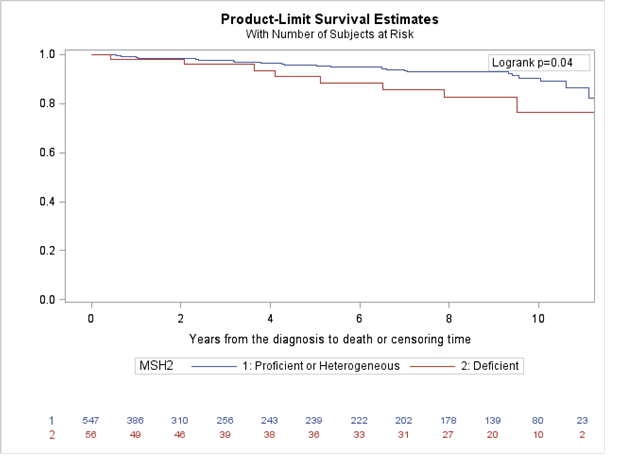

Supplement: Supplementary file 1 [file ijms-21-01461-s001.zip › Lopez et al - Int J Mol Sci - Supplementary Figure S4.tif]
